# The Microbiome of Aseptically Collected Human Breast Tissue in Benign and Malignant Disease

**DOI:** 10.1038/srep30751

**Published:** 2016-08-03

**Authors:** Tina J. Hieken, Jun Chen, Tanya L. Hoskin, Marina Walther-Antonio, Stephen Johnson, Sheri Ramaker, Jian Xiao, Derek C. Radisky, Keith L. Knutson, Krishna R. Kalari, Janet Z. Yao, Larry M. Baddour, Nicholas Chia, Amy C. Degnim

**Affiliations:** 1 Division of Subspecialty General Surgery, Mayo Clinic, Rochester, MN, USA; 2 Department of Health Sciences Research, Mayo Clinic, Rochester, MN, USA; 3 Center for Individualized Medicine, Mayo Clinic, Rochester, MN, USA; 4 Division of Surgical Research, Department of Surgery, Mayo Clinic, Rochester, MN, USA; 5 Department of Cancer Biology, Mayo Clinic, Jacksonville, FL, USA; 6 Department of Immunology, Mayo Clinic, Jacksonville, FL, USA; 7 Division of Infectious Diseases, Mayo Clinic, Rochester, MN, ville, USA; 8 Department of Physiology and Biomedical Engineering, Mayo Clinic, Rochester, MN, USA.

## Abstract

Globally breast cancer is the leading cause of cancer death among women. The breast consists of epithelium, stroma and a mucosal immune system that make up a complex microenvironment. Growing awareness of the role of microbes in the microenvironment recently has led to a series of findings important for human health. The microbiome has been implicated in cancer development and progression at a variety of body sites including stomach, colon, liver, lung, and skin. In this study, we assessed breast tissue microbial signatures in intraoperatively obtained samples using 16S rDNA hypervariable tag sequencing. Our results indicate a distinct breast tissue microbiome that is different from the microbiota of breast skin tissue, breast skin swabs, and buccal swabs. Furthermore, we identify distinct microbial communities in breast tissues from women with cancer as compared to women with benign breast disease. Malignancy correlated with enrichment in taxa of lower abundance including the genera *Fusobacterium, Atopobium, Gluconacetobacter, Hydrogenophaga* and *Lactobacillus*. This work confirms the existence of a distinct breast microbiome and differences between the breast tissue microbiome in benign and malignant disease. These data provide a foundation for future investigation on the role of the breast microbiome in breast carcinogenesis and breast cancer prevention.

Globally, breast cancer accounts for nearly one quarter of all cancers and is the leading cause of cancer death among women^1^. While there are established risk factors for breast cancer, at least 70% of breast cancer cases occur in women of average risk, and current prediction models provide poor risk discrimination for individuals^2^,^3^. Mounting evidence suggests that shifts in microbial assemblages are linked to cancer development and aggressiveness while eradication of the causative organism or restoration of the normal microbiota may reverse this process^4^,^5^,^6^,^7^,^8^,^9^. Our group has recently identified immune effectors in breast lobules, with cytotoxic T lymphocytes and dendritic cells intimately associated with normal breast epithelium^10^. Since mucosal immune systems develop as a direct result of microbial exposure, the presence of immune effectors within the complex microenvironment of the breast is suggestive of a breast microbiome.

In cancer, the microbiome has been implicated at a variety of body sites including stomach, colon, liver, lung, and skin with the strongest linkage thus far with gastrointestinal tract carcinogenesis, notably gastric cancer and lymphoma (*Helicobacter pylori*) and colon cancer (*Fusobacterium*)^5^,^8^,^11^,^12^,^13^,^14^. The distinct nature of the microbiome of each body niche suggests a similar organ specificity to microbial effects on inflammation and carcinogenesis^14^. Work with 3,2′-dimethyl- 4-aminobiphenyl hydrochloride (DMAB)-treated mice suggests a microbial role in breast tumorigenesis, but this has not been examined in humans^15^. More recently, 16S rDNA analysis has shown that milk samples from lactating women have diverse bacterial communities, but it is unknown whether these also are present in breast tissues^16^. Interestingly, the milk microbiome seems to recapitulate some risk features that are associated with breast cancer. For instance, the breast milk microbiome appears to differ from other human niches and a different, less diverse bacterial community has been observed in obese versus lean mothers^17^. Additionally, the gut microbiome influences estrogen metabolism via the enterohepatic circulation^18^,^19^. These data and the association of obesity, estrogen levels and inflammation with breast cancer risk provide a rationale for exploring a cancer-associated microbiome in breast tissue. Past studies have cultured bacteria from intraoperatively-obtained breast tissue, consistent with the presence of endogenous bacteria^20^,^21^. However, potential confounding factors, such as contamination from the skin, were not explored and genomic technology for testing was not available.

The lack of data on the microbiome of sterilely obtained human breast tissue in benign and malignant disease states analyzed with paired skin tissue samples by a culture-independent molecular genetic approach prompted this investigation. We hypothesized a resident microbiome in human breast tissue that is distinct from that of the overlying skin. We further explored differences in the microbiome of human breast tissue from women with benign and malignant disease in order to evaluate a potential role for a breast microbiome in carcinogenesis.

## Results

### Breast Tissue is a Distinct Microbiome Niche

**Figure 1:**
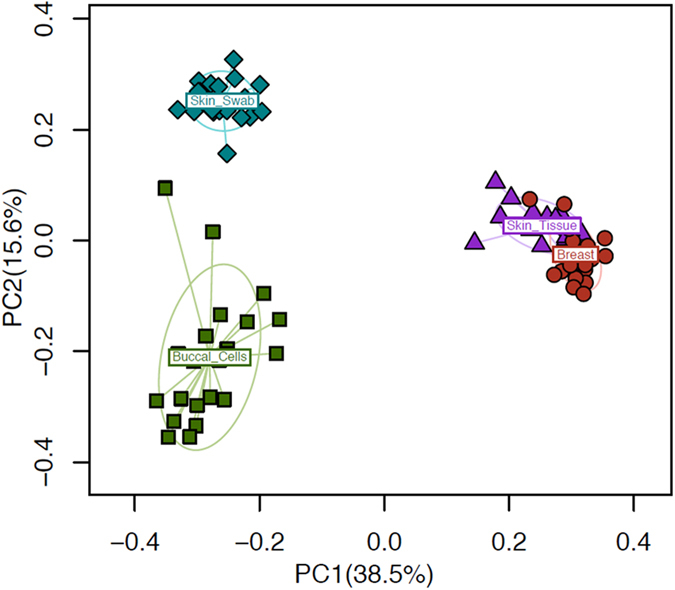
Ordination plot of samples from 33 women shows a distinct clustering pattern of different sample types (breast tissue, breast skin tissue, skin swab and buccal swab). The microbiota samples are embedded in the two-dimensional space based on the first two principal coordinates (PCs) from PCoA on the unweighted UniFrac distance. The percentage of explained variability of each PC is indicated on the axis. Each point represents a sample and is colored by sample types (blue diamonds - skin swab, green squares - buccal swab, purple triangles - breast skin tissue, red circles - breast tissue). The ellipse reflects the probability distribution of each sample type.

Principal coordinates analysis from 33 patients (whose demographic and clinical characteristics are described in Table 1) shows that sterilely-obtained breast tissue has a distinct microbiome. No patient had clinical evidence of infection at the time of operation nor developed a postoperative infection. Rarefaction curves to explore the sampling depth in the breast tissue specimens demonstrated sufficiency of reads at a median of 126,764 reads per sample (range: 28,549–335,363). A total of 1,460 operational taxonomic units (OTUs) have been detected for these samples (median: 454 OTUs per sample, range: 158–822). As shown in Fig. 1, the breast tissue microbiome is clearly distinguishable from that of the skin tissue, surface skin swab, and buccal swab samples (details of skin swab and buccal swab microbiota are provided in Supplemental Fig. 1). The microbiota of the breast tissue and the overlying breast skin tissue differ greatly from the buccal microbiome and that of the surface skin microbiome assessed by skin swab after antiseptic surgical prep. The microbiota of breast tissue and overlying breast skin tissue are closer to each other than to the buccal or skin swab communities but remain distinct.

### The Microbiome of Breast Tissue and Skin Tissue are Distinct

**Figure 2:**
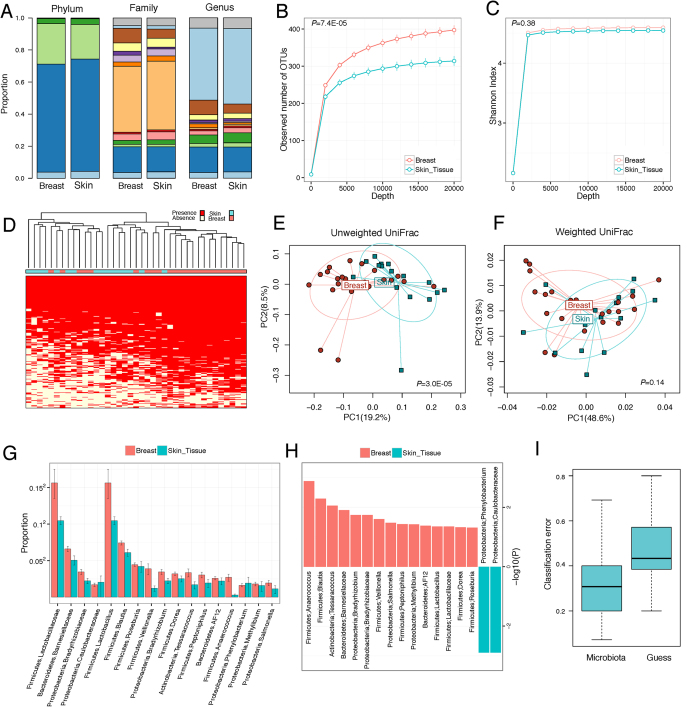
The microbiota of breast tissue is distinct from skin tissue in rare bacterial lineages. (**A**) Barplots of the taxonomic profiles of the breast and skin tissue microbiota at phylum, family and genus level for taxa with a relative abundance >0.5%. (**B**,**C**) Rarefaction curves compare the two alpha-diversity measures (observed OTU number (**B**) and Shannon index (**C**)) between the two tissue types. (**D**) Heat map shows the OTU presence and absence of all the tissue samples (column: samples, row: OTUs). The hierarchical clustering (top) is built based on the Euclidean distance of the OTU presence/absence profiles with a complete linkage. (**E**,**F**) Ordination plots show the clustering pattern of the two tissue samples based on unweighted (**E**) and weighted (**F**) UniFrac distance. (**G**,**H**) Differential taxa between breast and skin tissue microbiota based on a permutation test. Taxa with a nominal p value < 0.05 at the family and genus level are shown with their mean abundances in each tissue type (**G**) and their significance (**H**). Error bars represent standard error of the mean. (**I**) Boxplot compares classification error between a microbiota-based predictor (Left: Microbiota) and a predictor solely based on the majority class in the training set (Right: Guess). Random forest based on genus-level abundance is used to build the predictive model and 100 bootstrap samples are used for assessing the classification error.

More detailed analyses of paired sample sets from 32 patients comparing the breast tissue to the overlying skin tissue show that the breast tissue microbiome is distinct and separate from that of the breast skin tissue, primarily in rare bacterial lineages (Fig. 2). Evaluating the taxonomic profiles of the breast and skin tissue microbiota at phylum, family, and genus levels shows similar abundances of major taxa between breast skin tissue and breast tissue (Fig. 2A, detail in Supplemental Fig. 2). Alpha diversity analysis reveals that the breast tissue is richer in bacterial species than the skin tissue (P < 1e-4, Fig. 2B), while the species evenness (Shannon Index) is similar between breast tissue and skin tissue (P = 0.38, Fig. 2C). The increased species richness of the breast tissue microbiota is illustrated by the heat map (Fig. 2D), where more OTUs (mostly of low abundance) are observed in breast tissue microbiota. Beta diversity analysis reveals a highly significant difference in community membership between the breast and skin tissue microbiota, as indicated by unweighted UniFrac analysis (MiRKAT P < 1e-4, Fig. 2E), but the difference is not significant in weighted analysis (MiRKAT P = 0.14, Fig. 2F), indicating that the major difference lies in the rare or less abundant lineages^22^. To address potential concern about differential sequencing depth between breast tissue and skin tissues accounting for the differences observed in diversity, we adjusted for sequence depth in the model, in addition to rarefaction. After adjusting for sequence depth, the statistical significance for both alpha and beta diversity analysis was unchanged (i.e., significant differences remained in both alpha and beta diversity; data not shown). Evaluating differential taxa based on permutation testing also identifies differential taxa of low abundance (unadjusted P < 0.05, Fig. 2G,H), consistent with the beta-diversity analysis findings. These differentially abundant taxa were from the phyla Firmicutes, Actinobacteria, Bacteroidetes, and Proteobacteria. The distinct nature of the microbiota of breast versus skin tissue is further supported by a significantly lower classification error of a microbiota-based predictor (P < 1e-10, Friedman test), where we built a predictive model based on random forests using genus-level relative abundances as input, compared to a predictor solely based on the majority class in the training set (Fig. 2I). The result further supports that the breast tissue microbiota is different from the skin tissue microbiota. Despite differences in microbiota between skin tissue and breast tissue, we did observe a trend of greater similarity, though not significant, in paired breast and skin tissues from the same subject as compared to breast and skin tissue microbiota from different subjects (P = 0.13, permutation test, Supplemental Fig. 3).

### Breast Tissue Microbiome in Malignant versus Benign Disease States

To explore cancer-associated differences in breast tissue, we compared adjacent normal breast tissue samples from patients with benign breast disease without atypia (BBD-non-atypia) (n = 13) and invasive breast cancer (n = 15) whose characteristics are summarized in Table 2. A majority of the BBD-non-atypia subjects had proliferative disease (10/13 = 77%), while the remainder (3/13 = 23%) had nonproliferative disease. The invasive cancers were stage I in 10 patients (67%) and stage II in 5 (33%); two of 15 patients (13%) had a positive lymph node. The invasive cancers were all estrogen and progesterone receptor positive, and a minority (29%) were HER-2neu positive. Tumors were histologic grade I in 43% and grade II in 57%. These samples were randomized into two sequencing batches with equal representation of each disease state. Alpha- and β-diversity analyses revealed batch effects (P < 0.05 for all diversity measures). We thus adjusted batch effects in both diversity analysis and differential abundance analysis.

**Figure 3:**
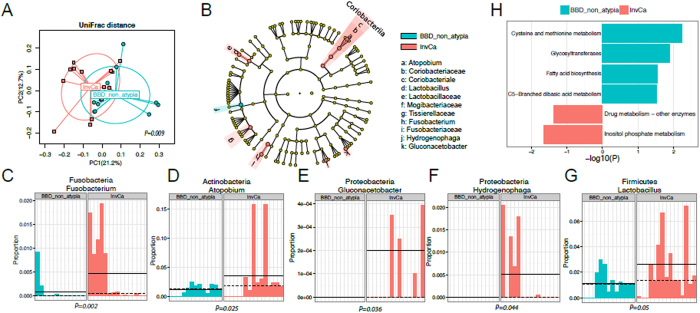
The microbiota of breast tissue adjacent to invasive cancer is distinguishable from that adjacent to benign disease (BBD-non-atypia). (**A**) Ordination plot based on unweighted UniFrac distance shows the clustering pattern of the breast tissue microbiota between the two disease states. (**B**) Differential taxa between the breast tissue microbiota of malignant and benign states based on a permutation test. Taxa with a nominal p value < 0.05 are highlighted on the cladogram with red and blue indicating increase and decrease in invasive cancer respectively. (**C–G**) Barplots show the abundances of the five differential genera between the two disease states. Each bar represents a sample. (**H**) The differential KEGG pathways with a nominal p value < 0.05 between the microbiota of the two states as revealed by PIRCRUSt analysis.

The microbiota of the normal breast tissue adjacent to invasive cancer was significantly different from that of normal breast tissue adjacent to benign disease (Fig. 3). An overview of taxonomic profiles shows the overall microbiota of breast tissue between the two disease states appear similar, dominated by Bacteroidetes and Firmicutes (Supplemental Fig. 4). Alpha diversity analysis shows no significant differences in observed OTU number nor in the Shannon index (Supplemental Fig. 5). However, beta diversity assessment with unweighted UniFrac distance (Fig. 3A) reveals the microbial community in breast tissue adjacent to invasive cancer is significantly different from that of women with benign disease (MiRKAT P = 0.009). However, the difference is not significant using the weighted UniFrac distance, indicating the major differences are mainly in rare and/or less abundant lineages. Utilizing a permutation test to assess differential taxa between the breast tissue microbiota in malignant and benign states demonstrates increased relative abundance in the following low-abundant genera in the breast tissue of women with invasive breast cancer: *Fusobacterium, Atopobium, Hydrogenophaga, Gluconacetobacter* and *Lactobacillus* (unadjusted P < 0.05, Fig. 3B and Supplemental Fig. 6). Barplots confirmed the differential abundances of the five differential genera between the two disease states (Fig. 3C–G). PICRUSt analysis^23^ demonstrates differential KEGG pathways between the microbiota of benign and malignant states, with benign tissues showing increased cysteine and methionine metabolism, glycosyltransferases and fatty acid biosynthesis, whereas cancerous tissue microbiota showed reduced inositol phosphate metabolism (unadjusted P < 0.05, Fig. 3H).

We reanalyzed the comparisons between benign versus malignant breast disease states including the few “intermediate” lesions of atypical hyperplasia (N = 3, classified as benign) and ductal carcinoma *in situ* (N = 2, classified as malignant). With this approach analyzing 33 samples, results were similar to the analysis of the 28 samples described above. Including the intermediate lesions, there were no observed differences in alpha diversity (P > 0.4). Similar to the N = 28 analysis, beta diversity analysis of the 33 samples showed significant differences in unweighted UniFrac analysis, indicating differences in rare and less abundant lineages. In addition, likely due to greater power from the slightly larger sample size, weighted UniFrac analysis also showed marginally significant differences. This suggests a potential widespread community change between benign and malignant breast tissues, although larger sample sizes are needed for confident characterization of critical differences in these tissues.

Since age and menopausal status vary significantly between disease states, either could potentially confound the identified associations. Thus we tested for menopause effects on the breast tissue microbiota using MiRKAT. This was not significant in both unweighted and weighted UniFrac distance (P > 0.5), indicating that the microbiota difference observed between disease states was not driven by differences in age/menopausal status.

## Discussion

We investigated the microbiome of sterilely obtained human breast tissue in women with benign and malignant breast disease. Two major findings from our study are that breast tissue obtained under surgically sterile conditions does indeed have its own distinct microbiome and that it is distinct from that of the overlying breast skin. The unique features of our study include (1) simultaneous collection of breast tissue, skin tissue and skin swab samples in the operating room under aseptic conditions and (2) comparison of the breast tissue microbiome in women with benign versus malignant disease. Our other key finding is that the background breast microbiome in women with malignant disease is notably different from the breast microbiome in women with benign disease. These data form the foundation for exploration of the core microbial community in breast tissue and microbial dysbiosis in association with health and disease including both cancer and infection. Dysbiosis of this intrinsic microbial community may contribute to cancer development and clinically apparent infection.

Previous work investigating the breast tissue microbiome using next-generation sequencing includes two studies. Xuan *et al*. compared breast tumor and unspecified negative control breast tissue from 20 patients with estrogen receptor-positive breast cancer^24^. One of the main confounding factors they discussed was the presence of potential contamination, which they were unable to address using retrospective collection of non-sterile formalin-fixed tissue. However, their results showed 1614 OTUs with 11 exhibiting differential abundance between tumor and normal tissue samples, with the predominant finding enrichment of *Sphingomonas* in normal tissue. The high number of OTUs they report possibly may be due to sample contamination. While this study assumes that contamination would be similar in the case and control samples, this study design makes it difficult to assess the true role of individual microbes in breast cancer. More recently Urbaniak *et al*. reported on 16S sequencing in unaffected breast tissue 5 cm from tumors in patients with malignant and benign tumors and in women undergoing breast reduction^25^. Also sterilely collected, they identify 121 OTUs in the breast microbiome and compared the relative abundance of taxa between women from Canada versus Ireland. The most abundant phyla in that report were Proteobacteria followed by Firmicutes, Actinobacteria and Bacteroidetes in descending order, consistent with our own findings. The investigators were unable to culture some of the most abundant taxa they identified, raising the question of whether some of these microbes were introduced from contaminated DNA rather than being intrinsic to the tissue samples. Further, the most prominent finding was a geographical difference between Canadian and Irish breast tissue microbiomes (although this may be artifactual as the samples were processed in different laboratories using different protocols and reagents), and there was no subsequent examination of benign versus malignant disease.

A second major finding from our study is that the breast tissue microbiome is distinct from that of the overlying skin tissue and has greater species richness. This would suggest that although the breast tissue microbiome may be derived from or contributed to by the skin microbiome, breast tissue has its own distinct environment and ecosystem. These differences may be attributable to differences in tissue microenvironments such as pH and oxygen levels which may facilitate relative dominance of certain taxa. Our data are supported by the findings from the Human Microbiome Project analyses that demonstrate the diversity and abundance of the signature microbes in each habitat varied among healthy subjects, with strong niche specialization both within and among individuals^26^.

Interestingly, while we found evidence using deep sequencing techniques that microorganisms are present in breast tissue samples obtained under aseptic conditions in the absence of clinical infection, this finding was suggested approximately 30 years ago in two studies after cultures of breast tissue obtained sterilely from the operating room resulted in positive cultures in up to 90% of samples^20^,^21^. In both studies, the most commonly cultured organism was coagulase-negative *Staphylococcus* and the most common anaerobe was *Propionibacterium acnes*, both predominantly skin bacteria that had potentially contaminated the surgical site. Indeed, this was the major weakness of these findings—one which we now have addressed using skin tissue and swabs. In the current study, samples of skin tissue from the incision edge were also evaluated in order to determine if the findings in breast tissue might be attributable to the skin organisms. With much more sensitive microbial detection techniques using genomic sequencing technology, we have confirmed that breast tissue indeed harbors a microbiome which is distinct from the microbiome of the overlying breast skin.

Perhaps our most intriguing finding in the present study was the observation of demonstrable differences in the breast tissue microbiome between women with benign versus malignant disease where we identified notable differences in beta diversity. Specific genus-level taxa that were significantly enriched in breast tissue from women with malignant disease include *Fusobacterium*, *Atopobium, Gluconacterobacter, Hydrogenophaga* and *Lactobacillus*. *Fusobacterium* has been reported in association with other epithelial malignancies including colon cancer and may act by secreting virulence factors as well as creating a pro-inflammatory environment that promotes carcinogenesis^4^,^5^,^6^,^7^,^27^. Therefore, we further investigated the functional role of these bacteria within these microenvironments. Using KEGG pathways as the basis for our analysis, we identified 6 differentially abundant pathways between benign and malignant disease states. In patients with malignant disease, pathways involving cysteine and methionine metabolism, glycosyltransferases, fatty acid biosynthesis and C5-branched dibasic acid metabolism were depleted. Interestingly, methionine dependence is a general metabolic derangement across multiple cancers and it is postulated that depleting methionine might reverse cancer progression, either using methioninase or a methionine-restricted diet^28^,^29^.

Our findings invite further study to define the origin of the breast tissue microbiome. Possible routes of bacterial access to breast tissue include passage from the skin via the nipple-areolar orifices, nipple-oral contact via lactation or sexual contact and bacterial translocation from the gut. Data supporting the first two hypotheses stems mainly from observations showing that the microbial composition of human breast milk in healthy lactating women contains many of the same bacteria found commonly in skin^16^ and shifts from a skin flora-dominant pattern in colostrum to an oral cavity-dominant pattern after 6 months of lactation^17^. In support of bacterial translocation from the gut, studies have shown that orally administered probiotics are recovered in the milk and treat the clinical infection lactational mastitis with better efficacy than orally administered antibiotics^30^. Further, in a clinical trial of orally administered *Lactobacillus salivarius* in late pregnancy, the rate and severity of lactational mastitis was significantly decreased^31^. Taken together, these studies demonstrate potential translocation of beneficial microbes from the gastrointestinal tract to the breast, although an alternative explanation of the protection from mastitis might be homing to the breast of anti-microbe B cells made in the gut. Additional indirect evidence of the possibility of gut to breast translocation comes from mouse studies showing increased rates of mammary gland carcinogenesis with enteral administration of certain pathogenic bacteria that appear to act in an immune cell-dependent fashion, although no data yet exists on the breast tissue microbiome in those experiments^32^. Future work to examine the relationship of multiple microbiome sites including gut, oral, skin and breast may help resolve some of these questions.

A limitation of our study is small sample size. Although we were able to detect an overall microbiota difference between tissue types and between disease states based on the MiRKAT test, the study was underpowered to identify specific differential taxa/KEGG pathways if a multiple testing correction procedure such as false discovery rate control is applied. Thus, in differential abundance analysis for both taxa and function data, we did not perform multiple testing correction in order to increase the power to identify true positives at the cost of a greater likelihood of false positives. A future study with a larger sample size is needed to identify with confidence a microbial signature for malignant breast tissue. The patients in this study with malignant disease all had estrogen receptor-positive tumors, as do 85% of breast cancer patients, but a different microbial signature may be present in the breast tissues of women with other biologic subtypes of breast cancer. To address the potential for contamination we did run negative controls as detailed in the methods. The significant differences that we identified in the microbial communities among breast tissue, skin tissue, skin swab and buccal swab samples suggest that contamination in collection, storage or processing does not account for the breast microbiome.

## Conclusions

Here we confirm the presence of a distinct breast tissue microbiome using culture-independent methods to analyze samples obtained and processed under aseptic conditions. Further, we show for the first time that this breast tissue microbiome is distinct from the overlying breast skin tissue, as well as from skin and buccal swab samples. In addition, we identified significant differences in the microbial composition of the breast tissue microenvironment in patients with benign versus malignant disease. While it is unclear whether small shifts in microbial communities or the presence of a virulent pathogenic strain or absence of a beneficial one might be responsible for promoting carcinogenesis, these findings are hypothesis-generating and support further investigation to identify a microbial risk signature for breast cancer and potential microbial-based prevention therapies.

## Methods

### Patients and Sample Procurement

With Mayo Clinic Institutional Review Board (IRB) approval (Mayo Clinic IRB#14-000815) and after written informed consent was obtained from all subjects, we enrolled 33 patients at least 18 years of age undergoing non-mastectomy breast surgery for cancer or benign disease into this prospective study. All research was performed in accordance with all relevant guidelines and regulations. Patients were excluded if they used antibiotics or systemic steroids or proton pump inhibitors^33^ within 14 days of operation, if there was clinical evidence of infection, any prior invasive cancer other than non-melanoma skin cancer, a documented chlorhexidine gluconate allergy, current pregnancy or lactation, prior organ or bone marrow transplant or had had a breast operation other than percutaneous needle biopsy within 3 months. At the time of enrollment, a buccal swab sample was obtained. Clinicopathologic data was obtained using a study case report form and entered into a REDCap database.

At operation, a standard sterile ChloraPrep skin prep and standard sterile draping was performed prior to skin incision. All but one patient received preoperative prophylactic intravenous antibiotics consisting of cefazolin in 31 cases and clindamycin in one case. The patient who did not receive preoperative antibiotics had a high-risk lesion and was not included in comparative analysis of the 28 patients with benign non-atypia versus malignant disease. In the operating room, once the targeted lesion was excised and margins were confirmed negative by intraoperative pathology assessment, and prior to wound closure, the surgeon obtained a skin swab from adjacent to the incision site (20 strokes with a sterile Dacron swab), a full thickness skin sample (2 mm by 20 mm or equivalent) excised from the edge of the skin incision and harvested one cubic centimeter of normal adjacent breast tissue from the margin of the lumpectomy cavity. Samples were placed immediately in sterile, study code-labeled tubes, snap frozen and stored at −80 °C in a dewar in the operating room and then transferred to −20 °C freezer until processing. The time of incision and sample acquisition, location of the study breast tissue and distance from the nipple was recorded on a study report form. The tissue immediately adjacent to the collected breast tissue sample was evaluated both intraoperatively with frozen section pathology and postoperatively with permanent sections which confirmed in all cases that there was no tumor or lesion present and was thus histologically normal. Four surgeons operating in four core operating rooms were trained and contributed samples to this study.

### DNA Extraction and 16S Sequencing

Prior to extraction, using sterile technique, frozen tissues were diced into small pieces and homogenized with a handhold homogenizer for 30 seconds in 100 μl of C1 buffer using the PowerSoil DNA Isolation Kit (MoBio Laboratories Inc. Cat. 12888). Extraction was performed following the manufacturer’s instructions. NEBNext Microbiome Enrichment Kit (New England BioLabs, NEB #2612S) was applied to remove the methylated host DNA in the genomic DNA for tissue samples. Quantitation of DNA was measured by Qubit 2.0 fluorometer (Life Technology) using Qubit dsDNA HS kit (Invitrogen Q32584). Blank controls were carried along throughout the entire extraction procedure for quality control.

To generate 16S amplicons, in a 50 ul reaction, typically 50 ng of gDNA was used as template, with 0.3 uM of V3-V5 barcoded primers targeting 357F and 926R of the bacterial 16S gene (5′AATGATACGGCGACCACCGAGATCTACACTATGGTAATTGTCCTACGGGAGGCAGCAG3′ and 5′CAAGCAGAAGACGGCATACGAGATGCCGCATTCGATXXXXXXXXXXXXCCGTCAATTCMTTTRAGT3′ respectively) and Kapa HiFi HotStart ReadyMix (Kapa Biosystem KK2602). Thermal cycling conditions were set as 95 °C for 3 minutes, following by 34 cycles of 98 °C 20 sec, 70 °C 15 sec and 72 °C 15 sec, then 5 minutes of extension at 72 °C. PCR products (385 bp) were verified via Agilent 2200 TapeStation System (Agilent Technology) with D1000 screen Tape (Agilent Technology Part No. 5067–5582) and purified with Agencourt AMPure Xp (Beckman Coulter) PCR purification procedure. All amplicons were quantified and then pooled to equalize concentrations for sequencing using Illumina MiSeq. Negative controls consisting of empty sterile storage tubes and swabs were processed for DNA extraction, amplified, and sequenced using the same procedures and reagents used for the tissue samples. There was no detectable amplification in the negative controls by qPCR.

### 16SrRNA Sequence Analysis

After sequencing, adapter-primer sequences were removed from reads as previously described^34^. In total, 15,156,581 reads passed quality control. Paired-end reads were analyzed according to the pipeline described in the IM-TORNADO bioinformatics pipeline^34^. Taxonomy was assigned against a Greengenes reference database (v13.5) and operational taxonomic units (OTUs) were assigned using a 97% identity threshold^35^,^36^. Taxonomic identification was manually checked using BLAST identifying one misclassification that we corrected in the downstream analysis. The sequencing depth of the negative controls and samples, as well as for BBD samples versus invasive cancers, is illustrated in Supplemental Fig. 7, and a barplot of taxonomic profiles of the negative control buccal and skin swabs at the phylum, family and genus level are shown in Supplemental Fig. 8.

### Statistical Analysis

#### α-diversity and β-diversity

To compare the microbial communities between groups (e.g. different tissue types and disease states), we summarized microbiota data using both α-diversity and β-diversity measures. Two α-diversity metrics were used, the observed OTU number and the Shannon index. The observed OTU number reflects species richness, whereas the Shannon index places more weight on species evenness. β-diversity, by contrast, indicates the shared diversity between bacterial populations in terms of ecological distance; different distance metrics provide distinctive views of community structure. Two β-diversity measures, unweighted and weighted UniFrac distances, were calculated using the OTU table and a phylogenetic tree (with the “GUniFrac” function in the R package GUniFrac)^22^. The unweighted UniFrac reflects differences in community membership (i.e., the presence or absence of an OTU), whereas the weighted UniFrac mainly captures differences in abundance. To reduce the potential confounding effect due to uneven sampling, we rarefied the OTU table to a sequencing depth of 20,000 per sample for both diversity analyses. To assess the association with α-diversity, we fitted a linear regression model to the α-diversity metrics after rarefaction, adjusting for technical covariates such as sequencing batch if necessary. A Wald test was used to determine significance. To assess the association between with β-diversity measures, we used the recently proposed MiRKAT^37^, which is a kernel-based association test based on ecological distance matrices. MiRKAT also allows easy adjustment of covariates such as sequencing batch. To further address the potential concern about differential sequencing depth between groups (Supplemental Fig. 7), we adjusted the sequence depth in the model, in addition to rarefaction. Ordination plots were generated using principal coordinate analysis as implemented in R (“cmdscale” function in the R ‘vegan’ package).

#### Differential abundance analysis

We conducted differential abundance analysis at the phylum, class, order, family, and genus levels, and we filtered out taxa with prevalence less than 10% and a maximum proportion (relative abundance) less than 0.2% to reduce the number of the tests. To identify differentially abundant taxa while accommodating covariates (e.g., sequencing batch) and the non-normality of the count data, we used a permutation test in which a regular linear model was fitted, with taxa proportion data as the outcome variable. To reduce the effects of outliers, taxa proportion data was square-root transformed. Statistical significance was assessed using 1,000 permutations with the F-stat as the test statistic. We reported differential taxa with unadjusted P < 0.05.

#### Predicting tissue type based on random forests

The machine learning algorithm random forests (RF)^38^ was used to predict tissue type based on the microbiota profile (genus-level relative abundance data) using default parameters of the R implementation of the algorithm. The RF algorithm, due to its non-parametric assumptions, can detect both linear and nonlinear effects and potential taxon-taxon interactions, thereby identifying the taxa that best discriminate between groups. We assessed the prediction accuracy based on bootstrapping and trained and tested the model using different divisions of the data set.

#### Functional data analysis

PICRUSt^23^ was used to infer the abundance of functional categories (KEGG metabolic pathways) based on the 16S rRNA data. Specifically, the input of PICRUSt is an OTU table built by a closed-reference OTU picking strategy, which involves a comparison to an existing reference (Greengenes v13.5). The output of PICRUSt is a count table of functional categories such as KEGG pathways constructed based on the functional content of each OTU. Rarefaction was not performed on the OTU table but singletons were removed before PICRUSt prediction. The predicted functional count table was normalized into relative abundances and differential abundance analysis was performed using the same permutation test that was used for the taxon analysis. Batch effects were adjusted in the model. We reported differential KEGG pathway with unadjusted P < 0.05, and differential abundance analysis was performed using the same permutation test that was used for the taxon analysis. All statistical analyses were performed in R 3.0.2 (R Development Core Team, Vienna, Austria).

## Additional Information

**How to cite this article**: Hieken, T. J. *et al*. The Microbiome of Aseptically Collected Human Breast Tissue in Benign and Malignant Disease. *Sci. Rep*. **6**, 30751; doi: 10.1038/srep30751 (2016).

## Supplementary Material

Supplementary Information

## Figures and Tables

**Table 1:** Patient, Tumor and Sample Collection Characteristics.

Variable	Total (N = 33)
**Age**, ***years***	
Median (Range)	60 (33–84)
**Menopausal status**, **n (%)**	
Pre-menopause	9 (27.3%)
Peri-menopause	2 (6.1%)
Post-menopause	22 (66.7%)
**BMI category**, **n (%)**	
18.5–24.9	7 (21.2%)
25–29.9	14 (42.4%)
≥30	12 (36.4%)
**Smoking status**, **n (%)**	
Current smoker	0
Former smoker	12 (36.4%)
Never smoker	21 (63.6%)
**Diabetes mellitus**, **n (%)**	
No	29 (87.9%)
Yes, NIDDM	4 (12.1%)
**Prior ipsilateral breast surgery**, **n (%)**	
No	32 (97.0%)
Yes	1 (3.0%)
**Parity**, **n (%)**	
0	6 (18.2%)
1–3	22 (66.7%)
>3	5 (15.2%)
**Breastfed ever**, **n (%)**	
No	14 (56.0%)
Yes	11 (44.0%)
*Missing*	8
**Family history of breast cancer**, **n (%)**	
No	21 (63.6%)
Yes	12 (36.4%)
**Distance of specimen from nipple**, **n (%)**	
≤2 cm	5 (15.2%)
>2 cm and ≤5 cm	12 (36.4%)
>5 cm	16 (48.5%)
**Time from incision to sample collection**, ***minutes***	
Median (range)	66 (30–206)
**Final diagnosis**, **n (%)**	
Benign Disease	16 (48.5%)
Cancer	17 (51.5%)

**Table 2:** Patient Characteristics by Benign (Non-Atypia) versus Malignant (Invasive Cancer) Disease Status.

Variable	Total (N = 28)	Invasive Cancer (n = 15)	Benign non-atypia (n = 13)	p-value^1^
**Age**				0.001
Median (Range)	60 (33–84)	75 (44–84)	49 (33–70)	
**Menopausal status**, **n (%)**				0.02
Pre-menopause	8 (28.6%)	2 (13.3%)	6 (46.2%)	
Peri-menopause	2 (7.1%)	0	2 (15.4%)	
Post-menopause	18 (64.3%)	13 (86.7%)	5 (38.5%)	
**BMI category**, **n (%)**				0.17
18.5–24.9	6 (21.4%)	4 (26.7%)	2 (15.4%)	
25–29.9	13 (46.4%)	8 (53.3%)	5 (38.5%)	
≥30	9 (32.1%)	3 (20.0%)	6 (46.2%)	
**Smoking status**, **n (%)**				0.28
Former smoker	10 (35.7%)	4 (26.7%)	6 (46.2%)	
Never smoker	18 (64.3%)	11 (73.3%)	7 (53.8%)	
**Diabetes mellitus**, **n (%)**				0.63
No	25 (89.3%)	13 (86.7%)	12 (92.3%)	
Yes, NIDDM	3 (10.7%)	2 (13.3%)	1 (7.7%)	
**Prior ipsilateral breast surgery**, **n (%)**				0.26
No	27 (96.4%)	14 (93.3%)	13 (100%)	
Yes	1 (3.6%)	1 (6.7%)	0	
**Parity**, **n (%)**				0.76
0	4 (14.3%)	2 (13.3%)	2 (15.4%)	
1–3	19 (67.9%)	10 (66.7%)	9 (69.2%)	
>3	5 (17.9%)	3 (20.0%)	2 (15.4%)	
**Breastfed ever**, **n (%)**				1.0
No	12 (57.1%)	8 (57.1%)	4 (57.1%)	
Yes	9 (42.9%)	6 (42.9%)	3 (42.9%)	
*Missing*	7	1	6	
**Family history of breast cancer**, **n (%)**				0.49
No	17 (60.7%)	10 (66.7%)	7 (53.8%)	
Yes	11 (39.3%)	5 (33.3%)	6 (46.2%)	
**Distance of specimen from nipple**, **n (%)**				0.53
≤2 cm	4 (14.3%)	2 (13.3%)	2 (15.4%)	
>2 cm and ≤5 cm	11 (39.3%)	5 (33.3%)	6 (46.2%)	
>5 cm	13 (46.4%)	8 (53.3%)	5 (38.5%)	
**Time from incision to sample collection**, ***minutes***				
Median (range)	69 (30–206)	82 (54–206)	52 (30–72)	<0.0001

^1^P-value was calculated using Wilcoxon rank-sum tests for continuous variables, chi-square tests for nominal variables, and Cochran-Armitage trend tests for ordinal variables.
