# Supplementary material for: The Microbiome of Aseptically Collected Human Breast Tissue in Benign and Malignant Disease
Source: Sci Rep. 2016 Aug 3;6:30751. doi: 10.1038/srep30751 (PMC4971513; doi:10.1038/srep30751)
Supplement: Supplementary Information [file srep30751-s1.doc]

**The Microbiome of Aseptically Collected Human Breast Tissue in Benign and Malignant Disease**

**Supplemental Information**

Tina J Hieken, MD

Jun Chen, PhD

Tanya L Hoskin, MS

Marina Walther-Antonio, PhD

Stephen Johnson, MS

Sheri Ramaker, RN

Jian Xiao, PhD

Derek C Radisky, PhD

Keith L Knutson, PhD

Krishna R Kalari, PhD

Janet Z Yao, MS

Larry M Baddour, MD

Nicholas Chia, PhD

Amy C Degnim, MD

**Supplemental Figure Legends**

**Supplemental Figure 1.** Barplot shows the taxonomic profiles of the buccal swab and skin swab microbiota at phylum, family and genus level. The taxa names are shown on the right. Only taxa with a relative abundance >0.5% are shown.

**Supplemental Figure 2.** Barplot shows the taxonomic profiles of the breast and skin tissue microbiota at phylum, family and genus level. The taxa names are shown on the right. Only taxa with a relative abundance >0.5% are shown.

**Supplemental Figure 3.** The skin tissue microbiota shows a trend of similarity with its paired breast tissue. **A**. Boxplot compares the unweighted UniFrac distances between paired breast and skin tissues, and the distances between unpaired breast and skin tissues, i.e., skin and breast tissue from different subjects. **B**. Histogram shows the null distribution of the differences in mean UniFrac distances (unpaired vs paired) under permutation, where the subject ID of a given tissue type is permuted. The observed difference (indicated by a red vertical bar) shows a trend of being larger than those under permutation (P = 0.131).

**Supplemental Figure 4.** Barplot compares the taxonomic profiles of the breast tissue microbiota between breast tissues in women with invasive cancer versus benign disease at phylum, family and genus level. The taxa names are shown on the right. Only taxa with a relative abundance >0.5% are shown.

**Supplemental Figure 5.** Rarefaction curves compare the two alpha-diversity measures (observed OTU number **(A)** and Shannon index **(B)**) between the two disease states.

**Supplemental Figure 6.** Differential taxa for the breast tissue microbiota of invasive cancer and benign disease patients based on a permutation test. Taxa with a nominal p value <0.05 at the family and genus level are shown with their mean abundances in each disease state **(A)** and their significance **(B)**. Error bars represent the standard error of the mean.

**Supplemental Figure 7.** Boxplots comparing the sequencing depth for samples based on sample type **(A)** and by the presence (InvCa) or absence (BBD) of invasive cancer within the breast **(B)**.

**Supplemental Figure 8.** Barplot shows the taxonomic profiles of the skin swab and buccal swab negative control microbiota at phylum, family and genus level. The taxa names are shown on the right. Only taxa with a relative abundance >0.5% are shown, with the others grouped in the gray shaded area as “other”.

**Supplemental Figures**

**Supplemental Figure 1**

**Supplemental Figure 2**

**Supplemental Figure 3**

**Supplemental Figure 4**

**Supplemental Figure 5**

**Supplemental Figure 6**

**Supplemental Figure 7**

**Supplemental Figure 8**
